# Emerging Anticancer Potentials of Goniothalamin and Its Molecular Mechanisms

**DOI:** 10.1155/2014/536508

**Published:** 2014-08-28

**Authors:** Mohamed Ali Seyed, Ibrahim Jantan, Syed Nasir Abbas Bukhari

**Affiliations:** ^1^Faculty of Pharmacy, Universiti Kebangsaan Malaysia (UKM), Jalan Raja Muda Abdul Aziz, 50300 Kualalumpur, Malaysia; ^2^School of Life Sciences, B.S. Abdur Rahman University, Seethakathi Estate, Vandalur, Chennai 600048, India

## Abstract

The treatment of most cancers is still inadequate, despite tremendous steady progress in drug discovery and effective prevention. Nature is an attractive source of new therapeutics. Several medicinal plants and their biomarkers have been widely used for the treatment of cancer with less known scientific basis of their functioning. Although a wide array of plant derived active metabolites play a role in the prevention and treatment of cancer, more extensive scientific evaluation of their mechanisms is still required. Styryl-lactones are a group of secondary metabolites ubiquitous in the genus* Goniothalamus* that have demonstrated to possess antiproliferative activity against cancer cells. A large body of evidence suggests that this activity is associated with the induction of apoptosis in target cells. In an effort to promote further research on the genus* Goniothalamus*, this review offers a broad analysis of the current knowledge on* Goniothalamin* (GTN) or 5, 6, dihydro-6-styryl-2-pyronone (C_13_H_12_O_2_), a natural occurring styryl-lactone. Therefore, it includes (i) the source of GTN and other metabolites; (ii) isolation, purification, and (iii) the molecular mechanisms of actions of GTN, especially the anticancer properties, and summarizes the role of GTN which is crucial for drug design, development, and application in future for well-being of humans.

## 1. Background

Cancer continues to be one of the major causes of death worldwide, despite technological advancements in various fields during the last two decades [1, 2]. Current estimates from the American Cancer Society and from the International Union against Cancer indicate that 12 million cases of cancer were diagnosed last year, accounting for 8.2 million deaths in 2012 worldwide; these numbers are expected to double by 2030, of which 62% arise in developing countries (27 million cases with 17 million deaths) [1–4]. As many as 95% of all cancers are caused by life style (lack of physical activity, tobacco, and alcohol use) and may take as long as 20–30 years to develop [5]. Due to its complex nature, treatment such as surgery, chemotherapy, photodynamic therapy (PDT), and radiation varies according to each type, location, and stage [6].

Medicinal plants are widely used by majority of populations as primary healthcare to cure various diseases and illnesses and have high an economic impact on the world economy [7, 8]. The increasing interest and scope of the drug of natural origin provides opportunities for its exploration, investigation, and utilization for biological activity [9–11] and particularly considered as cancer preventive or anticarcinogenic agents if they show good availability, low toxicity, suitability for oral application, and a vast variety of mechanisms of their action to prevent or at least delay and inhibit multiple types of cancer [12]. Various bioactive compounds from plant extracts have been experimentally tested to expand the clinical knowledge for its biological effects. As such, natural products have provided a continuous source of novel chemical structures in the development of new drugs and approximately 119 pure compounds isolated from plants are being used as medicine throughout the world.

## 2. Plants as Source of Anticancer Agents

Plants have a long history of use in the treatment of cancer. More than 3000 plant species have been reported to be involved in the development of anticancer drugs [13] and 60% of current anticancer agents have come from natural sources [14, 15] which include vinca alkaloids (vincristine, vinblastine, vindesine, vinorelbine), taxanes (paclitaxel, docetaxel), podophyllotoxin and its derivative (etoposide, teniposide), camptothecin and its derivatives (topotecan, irinotecan), anthracyclines (doxorubicin, daunorubicin, epirubicin, idarubicin), and others. Anticancer drugs target several cellular components and activate responses that go from cell repair to cell death [16, 17].

## 3. *Goniothalamus *spp

*Goniothalamus* is one of the largest genera of palaeotropical Annonaceae, with over 160 species distributed throughout tropical southeast Asia; the centre of diversity lies in Indochina and Western Malaysia [18]. Only 22 (13.7%) out of 160 species of* Goniothalamus* have so far been recognized and investigated out of which only five are medicinal, which are used to treat asthma, rheumatism, fever, malaria, cholera, stomachache, postpartum protective remedy, abortifacient, and insect repellent [19]. Various compounds have been isolated from* Goniothalamus* species, especially the low molecular weight phenolic styryl-pyrone derivatives as lactonic pharmacophore, quinoline, and isoquinoline alkaloid derivatives and phenanthrene lactones, terpenes, acetogenins, and flavonoids [20–25]. Few styryl-lactones extracted from* Goniothalamus *are (i) goniothalamin, (ii) altholactone, and (iii) cardiopetalolactone [26].

## 4. Bioactive Components of* Goniothalamus *spp

Acetogenins and styryl-lactones from* Goniothalamus *species have shown to be cytotoxic to different human tumor cell lines [27–29]. Other reported biological properties of some compounds are antifungal, antiplasmodial, antimycobacterial, insecticidal, antimalarial, anti-inflammatory, immunosuppressive, and inhibitor of platelet-activating factor (PAF) receptor binding activities [30, 31]. Currently, 100 styryl-lactones are available approximately which are either discovered from natural products or made as synthetic analogs. These compounds have been demonstrated to be cytotoxic with preference to kill cancer cells [28, 32–34].

It was reported [26] that GTN as the active constituent of the bark of* G. andersonii, G. macrophyllus* Miq., and* G. malayanus* and altholactone was characterized from* G. arvensis* Scheff. and from the* G. borneensis* Mat-Salleh [35, 36]. Cardiopetalolactone was characterized from the stem bark of* G. cardiopetalus *Hook.f. & Thoms. with altholactone, (iv) goniopypyrone, goniothalamin, (v) goniodiol, (vi) goniofufurone, and (vii) goniofupyrone [37, 38]. Goniofufurone, goniopypyrone, goniothalamin, goniodiol, (viii) goniotriol, and (ix) 8-acetylgoniotriol were isolated from the roots of* G. griffithii *[21–23]. An isomer of altholactone and (x) (+)-isoaltholactone was isolated from stem bark of* G. malayanus*, and from the leaves of* G. montanus* J. Sincl. and the roots of* G. tapis* Miq. [39] whereas goniolactones were identified from the roots of* G. cheliensis* [40]. Digoniodiol, deoxygoniopypyrone A, goniofupyrone, goniothalamin, deoxygoniopypyrone A, gonodiol-8-monoacetate, and gonotriol (xi) were characterized from the aerial parts of* G. amuyon*, collected in the southern part of Taiwan near the coastal regions [25, 41–45]. The petroleum ether extract of the stem bark of* G. sesquipedalis* collected in Bangladesh yielded 5-isogoniothalamin oxide [44] and 5-acetyl goniothalamin (xii) was characterized from* G. uvaroides* King collected in Bangladesh [34] and Chen et al. [46] isolated howiinol A from* G. howii* Merr. (xiii). The mode of cytotoxic action of styryl-lactone is described subsequently.

## 5. Isolation and Purification of Goniothalamin

**Figure 1:**
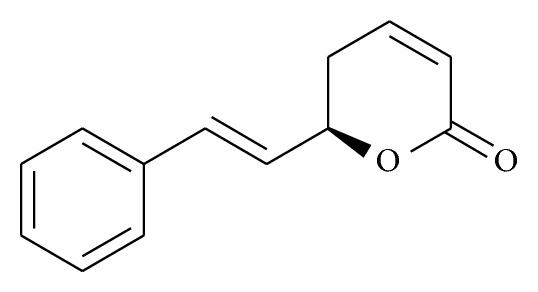
Chemical structure of goniothalamin.

Styryl-lactone GTN (Figure 1) was first isolated in 1972 [26, 47] since then it was subjected to extraction, isolation, and characterization. In most cases, the extracts were prepared by hot and cold extraction methods, that is, Soxhlet and percolation techniques, respectively. The crude methanol extracts were obtained by removing the solvent under reduced pressure and the yields were calculated based on dry weight. Bioactive compounds were isolated using various chromatographic techniques (VLC, column chromatography, Prep-TLC, etc.). The structures of bioactive compounds were also elucidated using spectroscopic techniques (1D, 2D NMR spectroscopy, FTIR, UV, mass spectrometry, etc.). Chromatographic fingerprint (HPLC) and spectrophotometric fingerprinting (ATR-FTIR) analyses with reference markers were also carried out on the plant extract. Briefly, the herbs were ground to powder, extracted in MeOH by ultrasonication for 30 min, and filtered. The chromatographic system consists of a HPLC equipped with a secondary pump, a diode-array detector, an autosampler, and a column compartment, a C18 column packed with 5 *μ*m diameter particles. A suitable solvent system was used for extraction process, for example, trifluoroacetic acid and acetonitrile was used with a linear gradient elution. Analytical technique using HPLC-DAD was developed and used to quantify the bioactive components of each extract as marker compounds. Preparation of the herb and the HPLC setup varied as per individual laboratory set up [48, 49].

## 6. Synthesis of Goniothalamin 

Due to its diverse pharmacological properties, GTN gained huge interest from researchers because several successful approaches have been adopted for its synthesis [50–54]. The absolute configuration in the pyran-2-one moiety has generally been secured from chiral starting material, asymmetric allylboration of aldehydes with *β*-allyldiisopinocampehylborane [50, 55, 56], or through asymmetric reduction using enzymes or microorganisms [51, 53, 54, 57–61]. De Fátima and Pilli [51] reported the syntheses of GTN via catalytic asymmetric allylation of α-benzyloxyacetaldehyde, followed by ring-closing metathesis and Wittig olefination, and via catalytic asymmetric allylation of trans-cinnamaldehyde, followed by ring-closing metathesis [62]. Gruttadauria et al. [54] along with coworkers reported that the high-yielding three-step synthesis of GTN involves an enzymatic kinetic resolution in the presence of vinyl acrylate followed by ring-closing metathesis [54]. GTN has been synthesized by lipase catalyzed resolution of (1*E*)-1-phenylhexa-1, 5-dien-3-ol using vinyl acrylate as acyl donor followed by ring-closing metathesis of the formed (1*R*)-1-[(*E*)-2-phenylvinyl] but-3-enyl acrylate. The unreacted alcohol from the resolution, (1*E*, 3S)-1-phenylhexa-1, 5-dien-3-ol, was esterified nonenzymatically and used for synthesis of GTN [53]. Das et al. [63] reported that the stereo selective total synthesis of GTN is achieved via a common intermediate. The synthesis employed the reduction of a propargyl ketone and olefin cross-metathesis as the key steps [63]. Fournier et al. showed that the diastereoselective [2+2]-cycloaddition of *β*-silyloxy aldehydes with trimethylsilylketene followed by HF-induced translactonization is a useful short method for the efficient synthesis of α, *β*-unsaturated-*δ*-lactones [64].

## 7. Mechanism of Action

### 7.1. Cytotoxic Activity against Cancer Cells

**Figure 2:**
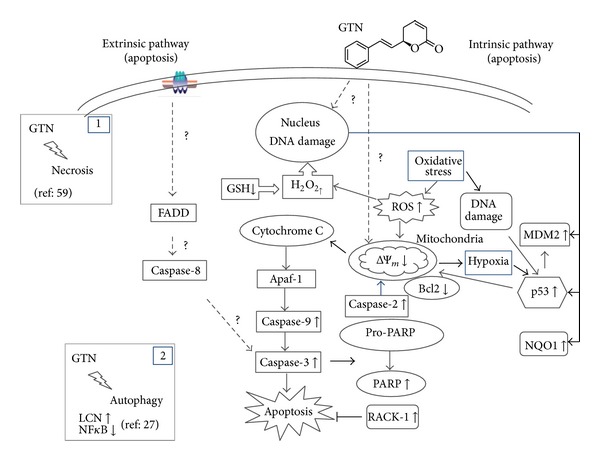
Schematic representation of mechanism of action of goniothalamin (GTN) in cancer cells. GTN mostly induces apoptosis either by DNA damage from oxidative stress where GTN decreases GSH level and increases ROS production or direct effect on DNA. Alternatively, GTN may directly affect mitochondria leading to ROS production. The GTN induced cellular stress response leads to the upregulation of p53 as an initial signal for apoptosis. Once activated, the p53 protein can directly or via processing caspase-2 trigger the release of cytochrome c without loss of membrane potential. This is followed by caspase-9 and caspase-3 subsequently. GTN may also act directly on mitochondria or induce the upregulation of Fas/FasL but that needs to be further investigated.

GTN, a simple styryl-lactone has significant potential in the development of a cancer drug as it has been reported to possess a wide range of biological activities (Figure 2) including anticancer [34], anti-inflammatory [65], immunosuppressive, and apoptotic effects [21, 24, 28, 66–68]. GTN had been able to induce cytotoxicity in a variety of cancer cell lines including vascular smooth muscle cells (VSMCs), Chinese hamster ovary cells, renal cells [69–71], hepatoblastoma [72, 73], gastric, kidney cells, breast carcinomas, leukemia, Jurkat cells [67, 69, 74–84], hepatocellular carcinoma [85], lung cancer cells [86], oral cancer cells [87, 88], and HeLa cells [89, 90] but sparing the normal cells including blood cells [83].

Besides the above, GTN has been proved to be only cytotoxic to ovarian cancer cell line (Caov-3) without causing cell death in normal kidney cell (MDBK) when compared to tamoxifen or taxol treated cells [32]. In addition, GTN showed lower toxicity to normal liver Chang cell line as compared to doxorubicin (known chemotherapeutic drug) [72, 73]. On the other hand a study by [75] reported the antiproliferative activity of GTN in some solid tumor experimental model with no evidence of toxic effects in the animals after single and repeated doses.

### 7.2. Induction of Apoptosis

GTN initially induces DNA damage which subsequently leads to cytotoxicity primarily via apoptosis in VSMCs [78]. This finding indicates that apoptosis that had occurred on this method was previously described by Cohen [91] and Ren et al. [92] and others on HeLa cells [92, 93]. The above findings were confronted by Alabsi et al. [90] that GTN stimulate DNA fragmentation, a characteristic feature of apoptosis in HeLa cell line at 24, 48, and 72 h after treatment. DNA fragments reveal, upon agarose gel electrophoresis, a distinctive ladder pattern consisting of multiples of an approximately 180 base pairs subunit. DNA ladder formation is observed only when the extent of oligonucleosomal cleavage is prominent. Alabsi et al. [90] suggested that internucleosomal cleavage of DNA is likely to be in the later phase of apoptotic process [91, 94, 95]. Some evidence has indicated that GTN exposure can alter the membrane properties [67].

Apoptosis can be either caspase-dependent or caspase-independent [96, 97]. However, the mechanism of caspase-independent apoptosis was still poorly understood until recently. Caspase plays important roles in execution of apoptosis through either extrinsic or intrinsic pathways [33]. The ability of GTN to induce apoptosis via caspase-3 activation against hepatoblastoma (HepG2) cells, whereas in human Jurkat T-cells both caspases 3 and 7 activation is involved, which is totally absent in normal Chang liver cells [24] and caspases 3 and 7 in human Jurkat T-cells [81]. In this study, HepG2 and Chang cells were treated with GTN for 72 h and analysed by TUNEL and Annexin-V/PI staining. Furthermore, the postmitochondrial caspase-3 was quantified using ELISA and alteration of cellular membrane integrity and cleavage of DNA were also observed. On the other hand, postmitochondrial caspase-3 activity was significantly elevated in HepG2 cells treated with GTN after 72 h. These findings suggest that GTN induced apoptosis on HepG2 liver cancer cells via induction of caspase-3 with less sensitivity on the cell line of Chang cells. Besides the above, it was also shown that the executioner caspase-3/7/9 activity, not initiator caspase-8, was increased in low level, less than onefold at 6 hours and 24 hours of treatment with GTN as compared to untreated cells [90]. Previous study also reported that the sequential activation of caspase-9 but not caspase-8 leading to the downstream caspase-3 cleavage was observed in GTN-treated coronary artery smooth muscle cells (CASMCs) [79].

It has also been reported that GTN induced apoptosis in HL-60 and Jurkat cells via mitochondrial pathway [67, 82]. Thus, these findings suggested the insignificant role of caspase-8 as an initiator caspase. Caspase-8 is not essential in GTN induced apoptosis in HeLa cells. In order to rule out the possibility of caspase-8 involvement in GTN induced apoptosis, a detailed appropriate study is still required. de Fátima et al. [70] reported that R-GTN and S-GTN markedly downregulated Bcl2, an antiapoptotic protein, and also induced PARP cleavage by causing apoptosis in renal cancer cells. In this study, authors have also reported interestingly that S-GTN enhanced the expression of LC3; a typical marker of autophagy and NFkappaB was downregulated in S-GTN-treated cells. Overall, these results indicate that the antiproliferative activity of the two enantiomers of GTN on renal cancer cells involved distinct signaling pathways, apoptosis, and autophagy as dominant responses towards R-GTN and S-GTN, respectively. Also, it was reported that GTN treatment induces cell cycle arrest at G2/M level [33] and concentration dependent necrotic type of cell death [74]. However, most of the studies have reported that GTN induced cell death predominantly occurred through apoptosis mode only.

It has been reported that cytotoxic stress either from DNA damage or mitochondrial impairment leads to apoptosis via the intrinsic pathway [78, 98]. The intrinsic pathway involves the release of proapoptotic proteins including cytochrome* c* from the inner membrane of mitochondria to the cytosol leading to activation of caspase-9 [99]. Most of the styryl-lactones including GTN and altholactone induce oxidative stress in MDA-MD-231 breast cancer cells, and Jurkat and HL-60 leukemic cells leading to apoptosis [40, 92, 100]. Although previous work has demonstrated that GTN induces DNA damage in CASMCs, which subsequently leads to apoptosis induction [101] and this study hypothesizes that GTN-induced oxidative stress and DNA damage resulted in p53 upregulation which was stabilized by NQO1 leading to caspase-2-dependent mitochondrial-mediated apoptotic pathway. However, the mechanisms of oxidative stress induced by styryl-lactones have not been unraveled. Numerous studies have demonstrated that the oncoprotein Bcl-2 can inhibit apoptosis by inhibiting the release of cytochrome* c* and can also modulate oxidant induced apoptosis [102]. Since the discovery of the caspase-9 apoptosome complex [103], more recent studies have shown that the initiator caspase-2 also forms a complex with RAIDD, a death receptor molecule, and the p53 inducible death domain PIDD forming a PIDDosome complex [104]. Importantly, caspase-2 has been demonstrated in a variety of cell lines to be activated upstream of mitochondria in genotoxin-induced apoptosis. Cleavage of the proapoptotic Bcl-2 family member Bid by caspase-2 has been shown to be required for cytochrome* c* release suggesting a potentially crucial role for caspase-2.

Although a large body of evidence suggests that various plant metabolites exterted their potentials against many cancer types through their unique mechanism of action for example, vincristine inhibits microtubule assembly, inducing tubulin self-association into coiled spiral aggregates [105]. Etoposide, a topoisomerase II inhibitor [106, 107] causes the stabilization of the clevable DNA- topoisomerase II covalent complexes, preventing subsequent DNA religation and stimulate enzyme-linked DNA breaks [108]. The taxanes paclitaxel and docetaxel has shown antitumor activity against breast, ovarian, and other tumor types in the clinic trial. Paclitaxel stabilizes microtubules and leads to mitotic arrest [109]. In addition, the camptothecin derivatives irinotecan and topotecan have shown significant antitumor activity against colorectal and ovarian cancer, respectively [100, 110], by inhibiting topoisomerase I [111]. Despite the above development, the unequal distribution of cancer burden between the developing and developed world is still largely looking for a better and safer anticancer compound for human use. Based on the data obtained from both* in vitro* cell culture and few* in vivo* animal models, GTN has demonstrated its potential against cancers and proven its insignificant effects on normal cells (Table 1). Taken together, undoubtedly GTN is emerging as promising agent in anticancer drug development with potential applications in cancer chemotherapy.

## 8. Conclusion

In conclusion, styryl-lactones are a group of secondary metabolites ubiquitous in the genus* Goniothalamus* that has demonstrated to possess interesting biological properties. These findings revealed that* Goniothalamus *plants do possess anticancer activity in a selective manner towards several tumor cell lines and initiate them to undergo different mode of cell death mainly apoptosis. Although the anticancer activity of the potential biomarker of this herbal plant, GTN on multiple cancer cells was through its regulation on cancer cell cycle and apoptosis induction mediated via oxidative stress and caspases activation and the antimetastatic and antiangiogenesis effects observed in GTN treated cells and animal, indicate its potential in inhibiting the development of secondary tumour. Further investigations into the mechanism of anticarcinogenic, antimetastatic, antiangiogenesis, and apoptotic regulation properties of GTN against various in vivo cancer models are still required. This may create an opportunity for the compound not only to be designed and developed as anticancer agent, but also to be used as an adjuvant or immunomodulators for combination chemotherapy against cancer. However, the preliminary* in vitro *data is insufficient and less convincing due to its limitation as most of the experiments are done in an* ex vivo* environment outside an animal or human body. Thus, more* in vivo *studies using various experimental cancer animal models are needed to determine the pharmacological and toxicological data as well as antitumour effect of GTN. Due to its diverse pharmacological properties, this compound gained huge interest among researchers that lead to the cost effective approaches for its synthesis; hence, this activity will further strengthen the efforts to identify more pathways and therapeutic action of this compound before it enters into the next phase of development. Overall, this compound provides information on the safe use and effectiveness that is crucial for drug design, development, and application in future for well-being of human.

## Figures and Tables

**Table 1:** Mechanism of action of Goniothalamin (GTN) in various cancer cells and their molecular effects.

S. no	Cell line *(in vitro) *	Animals *(in vivo) *	Mode of cell death	Molecular targets/effects	References
1	786-0 (renal cells)	—	Cytotoxicity/apoptosis	NOS↑/BCL2↓	[27, 70]

2	786-0 (renal cells)	—	Cytotoxicity/autophagy	LC3↑/NF*κ*B↓	[27]

3	Jurkat T-cells	—	Cytotoxicity/apoptosis	Caspases 3 and 7↑, oxidative stress, DNA damage RACK1↑	[81, 82] [80] [70]

4	HepG2 (hepatoblastoma) Chang (normal cells)	—	Cytotoxicity/apoptosis No toxicity	Caspase-3↑ Sparing normal cells	[72, 73] [72]

5	HCC (hepatocellular carcinoma)	—	Cytotoxicity/apoptosis	ROS↑	[85]

6	Caov-3 (ovarian) Caov-3 (ovarian) MDBK (normal kidney cells)	—	Cytotoxicity/apoptosis Antiproliferative No toxicity	Caspase-3↑ bcl-2↓ and bax↑ Sparing normal cells	[32] [77] [80]

7	MCF-7, T47D, MDA-MB-231 (breast cancer)	—	Cytotoxicity/apoptosis	Cell cycle arrest/modulating redox status	[33, 89]

8	MCF-7 (breast cancer)	—	Cytotoxicity/necrosis	Membrane integrity loss	[74]

9	COR-L23 (large cell lung carcinoma)	—	Cytotoxicity	Good cytotoxic compound to cancer cells	[68]

10	NCI-H460 (human nonsmall cell lung cancer cells)	—	Cytotoxicity/apoptosis	DNA damage	[86]

11	Ca9-22 (oral cancer)	—	Cytotoxicity/apoptosis	DNA damage, ROS↑, ΔΨ↓	[88]

12	U251 (glioma)	—	Antiproliferative	Good cytotoxic compound to cancer cells	[65]

13	OVCAR-03 (ovarian)	—	Antiproliferative	Good cytotoxic compound to cancer cells	[65]

14	PC-3 (prostate)	—	Antiproliferative	Good cytotoxic compound to cancer cells	[65]

15	W7.2 T-cells	—	Cytotoxicity/apoptosis	DNA damage, RACK1↑	[70]

16	NCI-460 (lung, nonsmall cells)	—	Antiproliferative	Good cytotoxic compound to cancer cells	[65]

17	NSCLC lung cancer	—	Cytotoxicity/apoptosis	DNA damage, MMP-2 and MMP-9↓	[87]

18	UACC-62 (melanoma)	—	Antiproliferative	Good cytotoxic compound to cancer cells	[65]

19	HL-60 (leukemia)	—	Genotoxicity/apoptosis	Ψ↓, caspase-9↑	[67, 80] [84, 101]

20	U937 (lymphoma)	—	Cytotoxicity/apoptosis	ΔΨ↓, caspase-9↑	[84]

21	CASMC (coronary artery smooth muscle cells)	—	Cytotoxicity/apoptosis	Caspase-2↑, p53↑	[78, 79]

22	HeLa (cervical)	—	Cytotoxicity apoptosis	Good cytotoxic compound to cancer cells DNA damage, caspase-9↑	[80–82] [90]

23	HGC-27 (gastric)	—	Cytotoxicity	Good cytotoxic compound to cancer cells	[74, 80–82]

24	768-0 (kidney)	—	Cytotoxicity	Good cytotoxic compound to cancer cells	[80–82]

25	HT-29 (colon) LS174T (colon)	— —	Cytotoxicity/apoptosis	Cell cycle arrest at S-phase	[89] [68]

26	3T3 (normal fibroblast) ST3 fibroblast	— —	No toxicity Cytotoxicity	Sparing normal cells Kills MMP1 expressing cells	[89] [68]

27	PANC-1 (pancreatic cancer)	—	Cytotoxicity/necrosis	Loss of cell membrane integrity	[74]

28	CHO (Chinese hamster ovary)	—	Genotoxicity	Causing damage to DNA	[69]

29	K562 (chronic myelogenous leukemia)	—	Cytotoxic and anti-inflammatory	NF-*κ*B↓	[83]

30	Platelets (rabbits)	—	Inhibitory	Platelet activating factor binding	[31]

31	Ehrlich tumor cells	Balb/C mice	Cytotoxicity	Tumor regression	[65]

32	Blood and serum parameters	Long Evans rats	Cytotoxicity	Biochemical/hematology and histopathology evaluation	[47]
